# Argus T® versus Advance® Sling for postprostatectomy urinary incontinence: A randomized clinical trial

**DOI:** 10.1590/S1677-5538.IBJU.2015.0075

**Published:** 2016

**Authors:** João Paulo Cunha Lima, Antonio Carlos Lima Pompeo, Carlos Alberto Bezerra

**Affiliations:** 1 Departamento de Urologia, Faculdade de Medicina ABC, Santo André, SP, Brasil

**Keywords:** Suburethral Slings, Urinary Incontinence, Therapeutics

## Abstract

**Objective:**

To compare the results of two slings, Argus T® and Advance®, for the treatment of postprostatectomy urinary incontinence (PPUI). Material and Methods: From December 2010 to December 2011, 22 patients with PPUI were randomized as follows: 11 (mean age 62.09(±5.30)) underwent treatment with Advance® and 11 (mean age 62.55(±8.54)) with Argus T®. All patients were evaluated preoperatively with urodynamic testing, quality of life questionnaire (ICIQ-SF), voiding diary and 24-hour pad test. Exclusion criteria were: neurological diseases, severe detrusor overactivity and urethral stenosis. Evaluation was performed at 6, 12 and 18 months after the surgery. After implantation of the Argus T® sling, patients who experienced urine leakage equal to or greater than the initial volume underwent adjustment of the sling tension. Results were statistically analyzed using the Fisher’s test, Kolmogorov-Smirnov test, Friedman’s non-parametric test or the Mann-Whitney test.

**Results:**

Significant improvement of the 24-hour pad test was observed with the Argus T® sling (p=0.038) , With regard to the other parameters, there was no significant difference between the two groups. Removal of the Argus T® device due to perineal pain was performed in one patient (9%). Despite non uniform results, both devices were considered useful to improve quality of life (ICIQ-SF): Argus T® (p=0.018) and Advance® (p=0.017).

**Conclusions:**

Better results were observed in the 24h pad test and in levels of satisfaction with the Argus T® device. Both slings contributed to improve quality of life (ICIQ-SF), with acceptable side effects.

## INTRODUCTION

Post-prostatectomy urinary incontinence (PPUI) is a common complication of surgical treatment in patients with prostate cancer or benign prostatic hyperplasia. It’s occurrence has a negative impact on quality of life (QoL) and decrease the benefit of the treatment of primary disease (1, 2).

PPUI prevalence varies from 2.5% to 67%. This is due to the wide variation in studies, such as non-standardized definition, type of surgical technique, diagnostic assessment, patient selection and outcome measures (3).

Over the last years, suburethral slings (SUS) have been re-designed and attracted particular interest due to its promising and durable results (3), even in face of the higher good results of the Artificial Urinary Sphincter-AUS 800^®^ (USA).

Two SUSs were available in the Brazilian market at the time of recruiting for this research: Argus T^®^-(Promedon–Cordoba, Argentina) (4, 5) and Advance^®^ (American Medical Systems-Minnetonka, United States) (6).

Both devices are transobturatory slings used to treat mild to moderate PPUI. Their technical configurations are different, as well as their mechanism of action, but both manufacturers claim that their products are effective and safe (4-6). There are no comparative studies analyzing their effectiveness and rates of complications. We present a randomized clinical trial comparing the results of these two devices at intermediate (18 months) follow-up.

## OBJECTIVE

The objective of this study was to compare the results of the surgical treatment of PPUI with Argus T^®^ and Advance^®^ slings.

## MATERIALS AND METHODS

This study was designed to be a randomized clinical trial. Randomization was made by computer-generated table of random numbers at www.random.org, and patients were assigned to one of the two treatment arms: Argus T^®^ or Advance^®^.

From December 2010 to December 2011, patients with PPUI were recruited from the outpatient services at two institutions (one public and one private).

Inclusion criteria: patients with 50 to 80 years of age with PPUI for at least the past six months, regardless of the level of incontinence. Exclusion criteria: patients with urethral stricture untreated or treated for less than 6 months, severe detrusor overactivity (when involuntary bladder contractions, as identified by urodynamic evaluation, where thought to be the primary cause of incontinence), and neurological disorders associated with neurogenic bladder.

Clinical evaluation consisted of history taking, interview to collect data such as results of prior pathological examinations, classification of risk of prostate cancer progression, as proposed by D’amico (7), and adjuvant treatments.

To better determine the level and impact of incontinence, patients were also submitted to: urodynamic evaluation (conducted following the recommendations of the International Continence Society (ICS) (8);

QoL questionnaire (assessed using the Brazilian Portuguese version of the “International Consultation on Incontinence Questionnaire-Short Form”-ICIQ-SF) (9);

voiding diary completed for three days in order to determine functional capacity (median voided volume), number of episodes of urine leakage, number of urinations and volumes of fluid intake and voiding;

24 hours pad test, done as recommended by ICS: all pads used in 24 hours were stored in a bag under refrigeration and weighted; total weight in grams was recorded to estimate total urine leak.

Surgical techniques were applied as proposed by original authors and are described, in summary, below (4-6). All procedures were performed by the same surgeon: patients were submitted to regional anesthesia, in the lithotomy position and legs flexed at close to 90 degrees at the thigh level. Skin preparation was made by applying topical polvidone-iodine or chlorhexidine to the perineum, thighs, scrotum, penis and lower abdomen. Foley 16F urethral catheter was inserted. Prophylactic antibiotic therapy was started no more than two hours before surgery with 2g of intravenous cefazolin and continued for 24 hours, with 1g every eight hours. After patient dismissal, 500mg of cephalexin was prescribed to be taken orally, every six hours, for seven days (10).

For the Advance^®^ sling, the tape was placed over the spongious body of the bulbar urethra and under the bulbospongious muscle through a perineal incision and transobturator route. A cystoscopy was performed to verify the presence of any urethral lesions and to certify that the bulbar urethral lumen had collapsed due to compression from the mesh tape. Bladder was drained with a Foley catheter for 24 hours and patient was instructed to restrain from any physical activity for the next 45 days.

The Argus T^®^ sling was placed through a longitudinal perineal incision and a transobturatory route. The sling was positioned over the bulbospongiosus muscle. The tension applied was sufficient to interrupt a retrograde infusion of saline solution through a Foley catheter, in a column with 35-40cm of H_2_O, with occlusion of the urethral meatus. Bladder was drained with a Foley catheter for 24 hours and patients were instructed to restrain from any physical activity for 45 days. In case of readjustment, the same retrograde occlusion pressure was the goal with re-tensioning.

Patients were dismissed after removal of the Foley catheter and after first normal void; revaluation was scheduled for the 7^th^ and 30^th^ days after surgery. Follow-up visits at 6, 12 and 18 months were programed; they underwent the 24-hour pad test, and completed the quality of life questionnaire (ICIQ-SF) and voiding diary at each visit. We present here the results after 18 months.

Post operatively, patients in the Argus T^®^ sling group, who experienced urine leakage equal to or higher than the baseline, underwent sling adjustment to reinforce urethral compression.

Additionally, in order to further compare both Groups, we established the following criteria to consider the patient cured:

- Average number of incontinence episodes over 24 hours lower than two.
- Average number of pads per day, up to one.
- 24-hour pad test with urine leakage less or equal to 50g.
- Assessment of quality of life (ICIQ-SF), with score reduction of 80% or more.

Finally, at the end of 18 months, the following questions were made to the patient to assess the degree of satisfaction: a) If you could return in time, would you undergo the same surgery again? b) How satisfied are you with the results obtained? c) Would you recommend to a friend, the same surgery you did? The questions were answered by the patients using a visual scale of 1 to 10, where 10 was certainly yes or satisfied and 1 certainly no or dissatisfied. To aggregate data, we used the following criteria: 1 to 3 meant no, 4 to 7, uncertain, and 8 to 10, yes.

Study protocol was approved by Institutional Review Board (Research Ethical Committee).

## RESULTS

**Figure 1:**
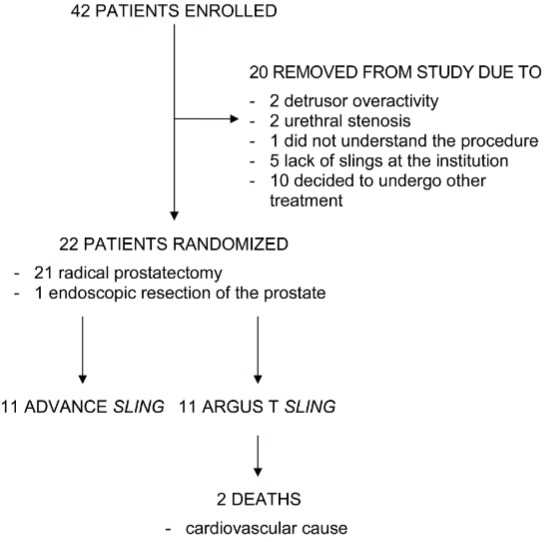
Study design.

Figure-1 shows the patient recruitment and randomization flowchart.

Randomization lead to homogenous groups regarding age, comorbidities, previous radiation therapy, previous treatment of urethral stricture, risk of recurrence as per the D’Amico classification system, number of urinations, number of pads used, number of urgency episodes, number of incontinence episodes, 24-hour pad test, incontinence questionnaire–short form ICIQ-SF, time interval between primary surgery and sling placement and urodynamic parameters (Table-1).

**Table 1:** Distribution of patients as per preoperative characteristics and group.

		Group		*p* ^(1)^

	Total	Advance^®^	Argus T^®^	

	n=22	n=11	n=11	
**Comorbidities**				0.361
High blood pressure	15	9	6	
Other	7	2	5	
**Radiotherapy**				0.586
Yes	4	3	1	
No	18	8	10	
**Urethral stenosis**				0.311
Yes	5	1	4	
No	17	10	7	
**Risk per D’amico classification**				0.750
Low	7	3	4	
Moderate	9	4	5	
High	6	4	2	
**24-hour pad test - preoperative**				0.658
< 100 g	1	1	0	
< 100-400 g	8	3	5	
< 400 g	13	7	6	

( 1 ) ^=^ Descriptive level of probability of the Fisher’s exact test

The length of surgery, 30 to 90 minutes, was similar in both groups.

During the postoperative follow-up, two patients in the Advance^®^ group experienced pain, which was relieved with analgesics; one had dehiscence of the surgical incision, and one had urinary retention due to sclerosis of the bladder neck after 12 months.

Complications were more frequent in the Argus T^®^ group. Two patients died of acute myocardial infarction: one, six months after the surgery, and one, 12 months afterwards. Both deaths were not related to immediate surgical complications, although they were clinically evaluated before sling surgery and had low cardiovascular risk. Three patients experienced urinary retention during 10 days and were dismissed with a Foley catheter in bladder. Four patients experienced prolonged pain: two improved with common analgesics, one is still taking medication with addition of a tryciclic antidepressant, and one had to remove the sling. Crural extrusion of the silicone column occurred in three patients. All cases were treated with antibiotics and removal of the extruded portion, with resolution of the problem without additional treatment.

Three patients underwent sling tension adjustment due to persistent leakage. Two were improved after 18 months and one remained with incontinence.

### Clinical Efficacy

Table-2 presents results regarding efficacy. No improvement on functional capacity, number of urinations, number of urgency episodes and incontinence episodes were observed in either group.

**Table 2:** Descriptive values of the variables analyzed, according to surgery and time point of evaluation (*).

Variance	Argus T^®^			Advance^®^		

	Baseline	18 months	p	Baseline	18 months	p
Functional capacity	**118.33**	**147.78**	0.407	**134.09**	**162.27**	0.726
	(±82.92)	(±105.57)		(±84.11)	(±11.31)	
QoL	**17.44**	**7.44**	0.018	**19.18**	**11.73**	0.017
	(±3.40)	(±6.98)		(±1.89)	(±8.36)	
24 hours pad test	**674.44**	**97.00**	0.038	**620.91**	**561.45**	0.386
	(±763.78)	(±218.60)		(±422.64)	(±890,09)	
No. of micturitions	**6.34**	**5.28**	0.325	**5.57**	**7.55**	0.421
	(±4.92)	(±4.14)		(±3.94)	(±4.93)	
No. of pad changes	**4.19**	**1.48**	0.066	**3.92**	**3.41**	0.167
	(±2.52)	(±2.79)		(±2.59)	(±4.22)	
Urgency episodes	**1.61**	**1.07**	0.416	**1.86**	**0.90**	0.400
	(±1.98)	(±2.25)		(±3.02)	(±1.96)	
Incontinence episodes	**4.56**	**2.67**	0.159	**3.62**	**8.51**	0.202
	(±3.61)	(±6.58)		(±3.13)	(±8.98)	

(*) Descriptive level of probability of the non-parametric Wilcoxon test

Better quality of life was observed in both groups. The Argus T^®^ group experienced a significant improvement in the 24-hour pad test, but the Advance^®^ group did not. Neither group had a significant improvement in the number of pad changes over 24 hours. However, results with the Argus T^®^ sling were very close to the level of significance.

The number of patients cured, based on the impact on incontinence episodes, pad changes, pad tests and quality of life scores was similar in the two groups (Table-3). The results of the quality of life questionnaire are shown in Table-4. The patients who underwent surgery with the Argus T^®^ sling had higher satisfaction rates than patients in the Advance^®^ group.

**Table 3:** Cure criteria assessment.

	Group		

Criteria	Advance^®^ (n=11)	Argus T® (n=9)	
	**n (%)**	**n (%)**	p*
Incontinence ≤ 2	5 (55.6%)	7 (77.8%)	0.620
Pads ≤ 1	5 (45.5%)	7 (77.8%)	0.197
Score QV ≥ 80%	3 (27.3%)	2 (22.2%)	1.000
PAD test 24 hours ≤ 50g	5 (45.5%)	7 (77.8%)	0.197

**Table 4:** Patient's impressions about the results after 18 months.

Advance :10 Patients	Yes	Doubt	No
Would undergo same surgery again	6	1	3
Degree of satisfaction (VAS)	4	2	4
Would indicate surgery to another person	7	0	3

Argus: 9 patients	Yes	Doubt	No

Would undergo same surgery again	9	0	0
Degree of satisfaction (VAS)	9	0	0
Would indicate surgery to another person	9	0	0

**VAS**=Visual analog scale

## DISCUSSION

There is still no optimal treatment for PPIU. Treatment within the first 12 months after surgery should be conservative since there is possibility of spontaneous restoration of urinary continence (11).

In regard to assessment of incontinent patients, many studies report the rates of improvement in the number of pads per day, but we know that pad changing is a personal decision and it varies too much from patient to patient. Therefore, this is not an accurate parameter to determine severity of incontinence (12, 13). For this reason, we assessed our patients with three major outcome measures: number of pads, voiding diary and QoL questionnaire. We expected to have more confident results with such broad evaluation.

When choosing a surgical technique, many authors prefer to use SUS in mild and moderate incontinence, reserving AUS for the most severe cases (14, 15). In our study, we decided to include patients with all degrees of severity because we believed that SUSs could be effective even in the most severe cases. In fact, if the patient wishes, there is no reason to not try a SUS, since an AUS still can be effective in the case of failed previous sling procedure. Nevertheless, patients should be advised not to expect the same results in severe cases, as in mild to moderate incontinence.

The basic difference between the Argus T^®^ and the Advance^®^ slings is that the first one is completely made of silicon and is adjustable, whereas the second one is a polypropylene mesh that is not adjustable. Moreover, the authors claim different mechanisms of action: the Argus T^®^ sling would correct PPUI by compressing the bulbar urethra (16, 17), whereas the Advance^®^ sling would act by repositioning the membranous urethra in the retropubic space which would increase its functional length and strengthen the sphincteric mechanism (3, 6, 15). Additionally, the authors who developed the Advance^®^ sling suggest that it is necessary to release the central tendon of the perineum and open the bulbospongiosus muscle to place the polypropylene mesh directly over the urethra. This action permits the elevation of the bulbar urethra and, consequently, the membranous urethra what leads to improvement of any residual continence mechanism.

On the other hand, the authors who developed the Argus T^®^ sling report that the silicone sling should be placed over the bulbar urethra, without opening the bulbospongiosus muscle. This provides better compression and lower risk of erosion. The effectiveness of the compression was determined by elevation of the closure pressure, measured by retrograde filling pressure (18, 19).

Unfortunately, observations from urodynamic examination after sling revealed that the only alteration detected in the parameters of the exam was the increase in VLPP with the Advance^®^ (20) sling. We really don’t know if any male sling led to elevation of voiding detrusor pressure.

The analysis of the efficacy parameters in our study revealed that, if we keep our focus on the quality of life criterion, both SUSs provide similar and significant improvement. However, if we direct our focus to more objective criteria, the Argus T^®^ sling had better results than the Advance^®^ (as observed by the pad tests results). From the patient’s point of view, this difference may or may not be important. For this reason, we looked at patient’s satisfaction rates in an additional questionnaire with a visual analog scale to assess their level of satisfaction with the results obtained. We then observed that most patients who underwent surgery with the Argus T^®^ sling are clearly satisfied with the treatment, whereas only a few satisfied patients in the Advance^®^ group. From a more technical point of view, using more strict criteria of cure, we notice that both slings are equally low effective. The sum up is that any analysis of effectiveness of the SUSs should be based on wide evaluation parameters, objective and subjective, in order to provide a better idea of the effects of the surgery. We notice that the literature does not follow this trend. Thus we think that our research adds new information by providing both patient subjective point of view and objective outcome measures.

In their early studies on Argus^®^, Romano et al. (5) reported: 73% of the patients were cured (no pads); 10% improved (one to two pads/day) and 17% failed and needed to use more than two pads/day (even after sling tension adjustment). In the follow-up on these patients, Romano et al. (16) reevaluated 47 of them, three years after sling implantation, observing that 66% (31 patients) were still cured, five needed to have the sling tension adjusted, 12.8% (6 patients) improved and 21% (10 patients) failed, showing that the results were long-lasting at the 36-month follow-up. In our study, three patients underwent sling tension adjustment, which allowed the restoration of continence and increased the success rates. We observed that the average number of pads/day decreased from 4.19 before the surgery to 1.48 after surgery, at 18 months. Despite being similar to the results reported by Romano et al. (16), these findings were not statistically significant. It should be stressed that the p value almost reached the level of significance (p=0.066), suggesting a trend. Or, it could only be devoid to our small sample size. This observation deserves continuing follow-up and augmentation of sample size to correct clarification. Additionally, we observed that 77.8% of our patients used a maximum of one pad per day after 18 months, similar to what was reported by these authors.

With regard to the complications associated with SUS implantation, Rehder et al. (15) monitored 156 patients who had undergone surgery with the Advance^®^ sling for 36 months and reported 109 complications: 50% cases of perineal pain, 9.6% temporary urinary retention, 4.4% dysuria, 5% perineal hematoma, 0.6% urinary infection, 0.6% surgical wound infection and one late complication (0.6%) from sling extrusion due to symphysite. In a study with 230 patients who underwent surgery with the Advance^®^ sling, Bauer et al. (14) also reported 23.9% of complications, 21.3% of which were transitory urinary retention, and only two patients, one with ileal neobladder and another with urethral lesion, continued to use intermittent clean self-catheterization. Other complications totaled 2.5% (0.4% surgical wound infection, 0.4% urinary infection, 0.4% persistent perineal pain) and only 1.3% of the cases needed to undergo new surgery (0.9% extrusion and 0.4% urethral perforation). We observed that rates of complications in both groups were similarly low and not very severe.

In our study, the following complications were recorded: two patients experienced transitory perineal pain which was resolved with painkillers, one had dehiscence of the surgical incision which was satisfactorily resolved and one patient, after 12 months, experienced urinary retention due to sclerosis of the bladder, treated with endoscopic internal urethrotomy, developing severe urinary incontinence.

With regard to the complications resulting from implantation of the Argus T^®^ SUS, in their initial results with 48 patients, Romano et al.(5) reported that 15% had transitory urinary infection, 21% had perineal pain that improved after six months, and 6% needed to have the sling removed (two due to urethral erosion and one due to infection). In the follow-up of these patients after three years, Romano et al. (16) reported that it was necessary to remove nine slings (19.1%), six of them due to erosion and three due to infection. Hubner et al. (21), in their experience with 101 patients who underwent surgery with an Argus T^®^ sling (retropubic approach), reported that after 2.1 years of follow-up there were 16 cases (15.8%) of sling removal due to erosion or infection. Additionally, they also observed that 15 patients (15%) experienced perineal pain which was resolved three months later with the use of regular painkillers. During the intraoperative period, there were 5 cases (5%) of minor bladder perforations.

An analysis of these studies reveals that complications arising from the surgery with the Argus T^®^ device are more frequent and severe than complications resulting from surgery with the Advance^®^ sling, requiring removal of the device in 6% to 35% of the cases. This could be due to the action mechanism of the Argus T^®^ sling, which probably exerts more pressure against the urethra and the surrounding tissues. Other factors, such as previous RT and urethral stricture, as well as prior treatment of PPUI, require further studies with larger samples to assess the relationship between risk factors and complications.

Our study has limitations and potential for bias. Our sample size was limited to the few slings provided to do this research. This significantly affects the power of data. We wait for funding to continue including patients to this series or starting another trial with new slings, once the Advance sling will be replaced by a new generation from the manufacturer. We cannot be sure if the differences found in results of each sling are real or occurred only by chance.

In summary, after comparing our results with the literature, we concluded that slings present promising success rates, with improved patient quality of life and satisfaction. Our study compared both slings, in similar populations and with fewer restrictions on inclusion, observing worse results with the Advance^®^ sling and more complications with Argus T^®^.

The new data we provide with this report is that the possibility of readjustment may be a considerable advantage for patients choosing sling for treatment of PPUI. The limitation of our study is the initial small sample size. We look forward to continue to recruit patients to enlarge our series and provide strongest evidence in the field.

## CONCLUSIONS

Better results were observed in the 24h pad test and in levels of satisfaction with the Argus T^®^ device. Both slings contributed to improve quality of life (ICIQ-SF), with acceptable side effects.
